# Transient Expression and Purification of Horseradish Peroxidase C in *Nicotiana benthamiana*

**DOI:** 10.3390/ijms19010115

**Published:** 2018-01-01

**Authors:** Suzanne M. Huddy, Inga I. Hitzeroth, Ann E. Meyers, Brandon Weber, Edward P. Rybicki

**Affiliations:** 1 Biopharming Research Unit, Department of Molecular and Cell Biology, University of Cape Town, Rondebosch 7701, South Africa; suzanne.botha@gmail.com (S.M.H.); Inga.hitzeroth@uct.ac.za (I.I.H.); ed.rybicki@uct.ac.za (E.P.R.); 2 Aaron Klug Centre for Imaging Analysis, University of Cape Town, Rondebosch 7701, South Africa; brandon.weber@uct.ac.za; 3 Institute for Infectious Disease and Molecular Medicine, University of Cape Town, Rondebosch 7701, South Africa

**Keywords:** horseradish peroxidase, transient expression, *Nicotiana benthamiana*, *Agrobacterium tumefaciens*, infiltration, recombinant protein

## Abstract

Horseradish peroxidase (HRP) is a commercially important reagent enzyme used in molecular biology and in the diagnostic product industry. It is typically purified from the roots of the horseradish (*Armoracia rusticana*); however, this crop is only available seasonally, yields are variable and often low, and the product is a mixture of isoenzymes. Engineering high-level expression in transiently transformed tobacco may offer a solution to these problems. In this study, a synthetic *Nicotiana benthamiana* codon-adapted full-length *HRP* isoenzyme gene as well as C-terminally truncated and both N- and C-terminally truncated versions of the *HRP C* gene were synthesized, and their expression in *N. benthamiana* was evaluated using an *Agrobacterium tumefaciens*-mediated transient expression system. The influence on HRP C expression levels of co-infiltration with a silencing suppressor (NSs) construct was also evaluated. Highest HRP C levels were consistently obtained using either the full length or C-terminally truncated HRP C constructs. HRP C purification by ion exchange chromatography gave an overall yield of 54% with a Reinheitszahl value of >3 and a specific activity of 458 U/mg. The high level of HRP C production in *N. benthamiana* in just five days offers an alternative, viable, and scalable system for production of this commercially significant enzyme.

## 1. Introduction

Peroxidases are a widely distributed class of enzymes found in animals, plants, and microbes. They catalyse the oxidation of various organic and inorganic substrates in the presence of hydrogen peroxide [1,2]. One of the most comprehensively studied group of plant peroxidases are those isolated from the roots of horseradish *Armoracia rusticana* [3], a hardy perennial herb cultivated mainly in the temperate regions of the world [4]. The roots contain numerous peroxidases or isoenzymes, as shown by Naatsaari et al. [5] who sequenced the *A. rusticana* transcriptome by NGS, and were able to identify 28 secretory peroxidases (isoenzymes). Known as horseradish peroxidases (HRPs), the enzymes or isoenzymes are grouped according to their isoelectric points (pI) into acidic (A), neutral (B and C), or basic (D and E) groups [6]. Of these, the isoenzyme C (HRP C; EC 1.11.1.7) is the most abundant, widely studied, and utilized isoenzyme [2,4]. HRP C is commercially significant, particularly as a reporter enzyme in colorimetric and chemiluminescent assays in molecular biology, and in medical diagnostic kits [4,7,8]. HRP C also has further uses as a reagent for organic synthesis and biotransformation, and in waste water treatment [4,6].

Mature HRP C is comprised of a single polypeptide of 308 aa, as determined by Welinder [9]. It has four disulphide bonds and nine potential N-glycosylation sites, eight of which are glycosylated, resulting in the mature HRP C having a total carbohydrate content of between 18% and 22% [4]. HRP C has a metal centre of iron (III) protoporphyrin IX (referred to as a haem group) and two calcium ions. This means that the total molecular mass (Mr) of HRP C is approximately 44 kDa [6].

The cDNA sequence encoding HRP C shows the presence of a hydrophobic N-terminal leader sequence of 30 amino acids, and a C-terminal extension of 15 amino acids [3,6]. Indeed, HRP C is initially synthesized as a preproprotein, and during maturation the N- and C-terminal extensions are removed. The N-terminal sequence is responsible for directing HRP C to the endoplasmic reticulum (ER), where it undergoes post-translational modifications, including disulphide bond formation, N-glycosylation, and haem and Ca^2+^ incorporation [10]. In eukaryotic systems, targeting of HRP C to the ER and subsequent glycosylation appears to be essential for activity and expression of the protein. This N-terminal extension has also been suggested to have a role in mRNA stability of the transcript, since in its absence, HRP C transcripts were not detected in transgenic tobacco plants [11]. The C-terminal extension is a C-terminal-dependent vacuolar sorting signal (ctVSS), directing secretion of HRP C to the vacuole [12]. Functional expression of HRP C lacking the C-terminal ctVSS has been successfully shown in tobacco [10,11,13], and such expression results in secretion of the protein into the apoplastic space, although this secretion is seemingly inefficient [11,13].

Commercially available horseradish peroxidases are usually purified from the roots of horseradish (*Armoracia rusticana*) [4]. Cultivated crops are, however, only available seasonally, and in many cases the HRP content is either low or variable, and subject to environmental conditions. Furthermore, obtaining a pure preparation of a single isoform is complicated, since the native plant contains many isoforms of the enzyme [14]. Engineering high-level expression of HRPs in alternative systems may offer a solution to these problems. Successful recombinant expression of HRP C has been attempted in a number of different systems, including *Escherichia coli*, *Saccharomyces cerevisiae*, *Pichia pastoris*, Sf-9 insect cells, transgenic tobacco, *Rachiplusia nu* larvae, and *Spodoptera frugiperda* larvae [2,7,8,13,15,16,17]. However, most systems produce only low yields, or there are certain problems associated with the recombinant protein, such as hyperglycosylation in yeast systems [7], and inactivity that requires in vitro re-folding when expressed in *E. coli* [13]. Levels attained in Sf-9 suspension cultures reached 41.3 mg/L [2], while levels in *R. nu* larvae reached 100 mg/kg [16] and 137 mg/kg in *S. frugiperda* larvae [17]. Research conducted on recombinant expression of HRP C in tobacco has been aimed more at HRP C as a plant peroxidase and its role in vivo in the plant defense system than in recombinant production [1,11,12,18,19]. However, Matsui et al. [13] report attaining levels of 3 mg/L after seven days expression in transgenic tobacco BY2 cultures. This equated to approximately 100 mg of HRP/kg of transgenic BY2 cells [13].

The advantages of using plant systems to produce heterologous proteins include the following: there is proper protein folding and processing comparable to other eukaryotic systems; lower raw material costs, meaning that there is a relatively low cost to producing large amounts of product, and easy scalability of the production process [20]. While plant expression technology is still often based on stably transformed plants or cells, transient expression systems have become much more popular in recent years, simply because of the convenience and speed of the systems. Large amounts of protein are attainable in just days, compared to the months required for transgenic expression [21,22]. A techno-economic analysis carried out by Walwyn et al. [23] has shown that large scale production of HRP C (>5 kg HRP C per year) by transient expression in *Nicotiana benthamiana* is economically competitive compared with the current method of extraction of HRP from horseradish. Thus, transient expression of HRP C in plants may offer a cheaper, more rapid, and reliable method for production of this commercially important enzyme, as well as providing a single isoenzyme.

In this study, we investigated the possibility of using a synthetic *N. benthamiana* codon-adapted *HRP C* gene for high level *Agrobacterium*-mediated transient expression in *N. benthamiana* plants. Full length *HRP C* encoding sequences, along with constructs for C-terminally truncated and both N- and C-terminally truncated versions of *HRP C*, were cloned into four different plant expression vector constructs to allow trafficking of HRP C to various cellular compartments (vacuole, apoplast, chloroplast, and ER). Expression levels of recombinant HRP C were compared with and without the addition of a silencing suppressor, and the one yielding the highest level was selected for further purification and activity testing.

## 2. Results

### 2.1. Construction of Recombinant HRP C Vectors

**Figure 1:**
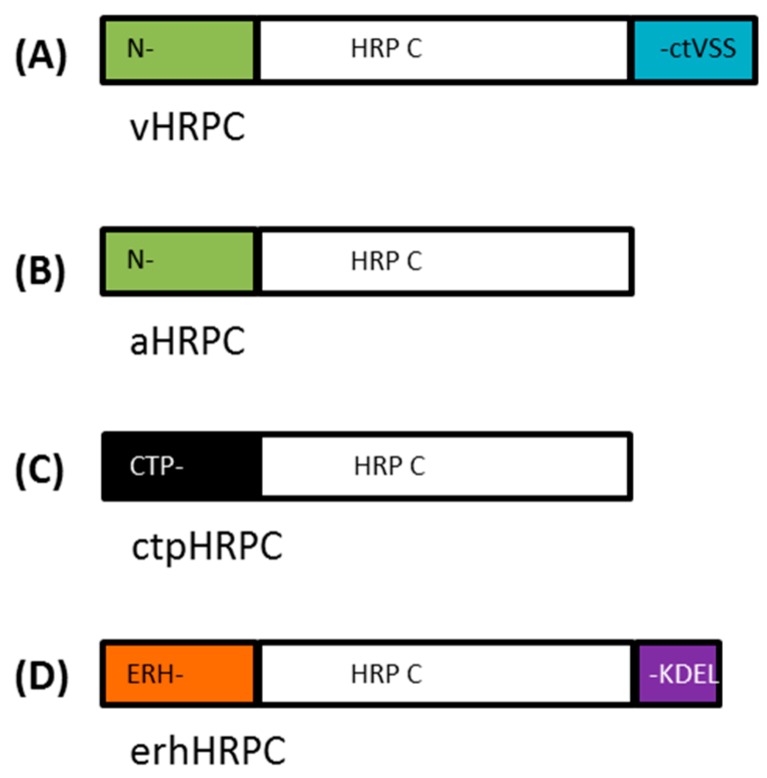
Engineered HRP C proteins used in this study. Schematic representation showing (**A**) the native HRP C (vHRPC), including the N-terminal signal peptide (N-) and C-terminal-dependent vacuolar sorting signal (ctVSS), which would result in secretion to the vacuole; (**B**) C-terminally truncated native HRP C (aHRPC), resulting in apoplastic secretion; (**C**) a N- and C-terminally truncated HRP C (ctpHRPC), including a chloroplast-transit peptide (-CTP) from the potato *rbcS1* gene, resulting in chloroplastic targeting; and (**D**) a N- and C-terminally truncated HRP C (erhHRPC), including a murine mAb24 heavy-chain signal peptide (ERH-) for ER targeting as well as an ER retention signal (-KDEL). Diagram is not to scale.

**Figure 2:**
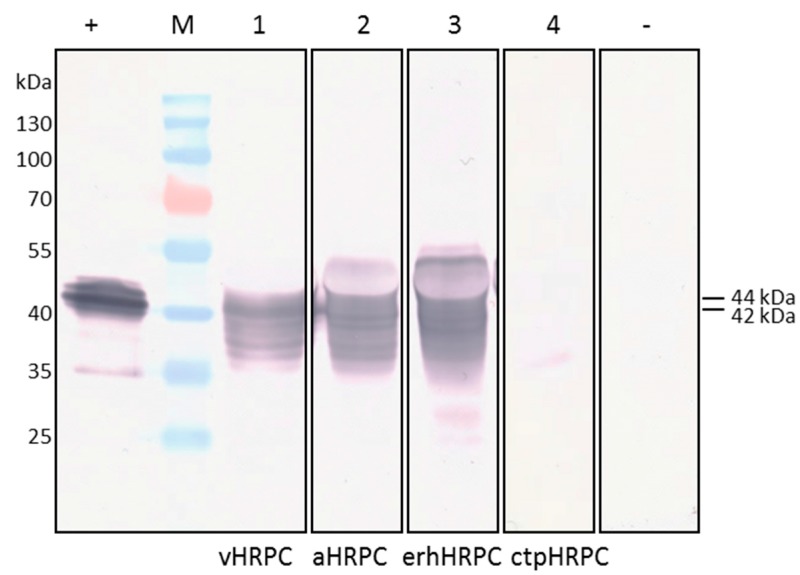
Western blot analysis of HRP C transient expression in *N. benthamiana* 5 days post infiltration. HRP C was detected using mouse polyclonal anti-HRP C. Crude plant extracts were analysed from plant tissue infiltrated with recombinant *Agrobacterium* strains carrying the following expression vectors pTRAc-HRPC (vHRPC) (1); pTRAc-HRPCΔC (aHRPC) (2); pTRAkc-ERH-HRPCΔNC (erhHRPC) (3); and pTRAc-CTP-HRPCΔNC (ctpHRPC) (4). Plants infiltrated with *Agrobacterium* strain LBANSs and sampled 5 days post-infiltration served as a negative control (−). Commercial Type I HRP C (400 mU) served as the positive control (+). Prestained protein ladder (M).

**Figure 3:**
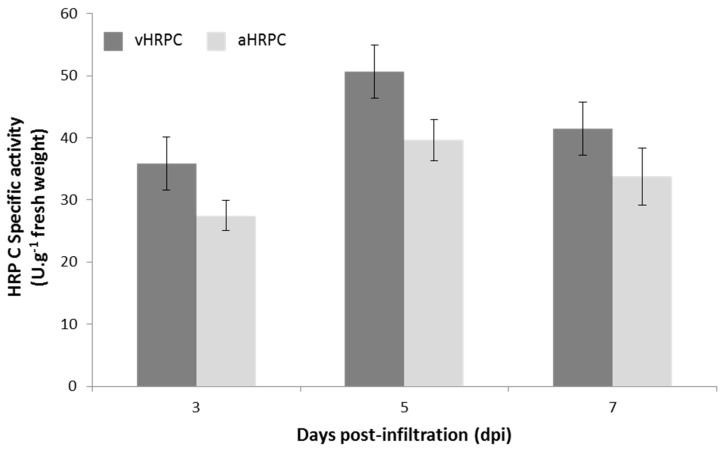
HRP C activity at different days post-infiltration with pTRAc-HRPC or pTRAc-HRPCΔC. Results are expressed as the average of 4 biological repeats ± standard error. 1 Unit of HRP C is defined as the amount of enzyme required to catalyse the conversion of 1 micromole of hydrogen peroxide per minute at 25 °C.

The components of the four different plant expression constructs made to target HRP C to four different cellular compartments are summarized in Table 1. These included the following: (1) pTRAc-HRPC encoding HRP C with its native N-terminal leader sequence for ER targeting and its native C-terminal-dependent vacuolar sorting signal (ctVSS) resulting in targeting of HRP C to the vacuole (vHRPC; Figure 1A); (2) pTRAc-HRPCΔC encoding HRP C lacking the C-terminal ctVSS resulting in targeting of HRP C to the apoplast (aHRPC; Figure 1B); (3) pTRAc-CTP-HRPCΔNC encoding HRP C lacking both its native N-terminal ER-targeting signal peptide and ctVSS but having a chloroplast-transit signal peptide sequence fused to its 5′ terminus resulting in targeting of HRP C to the chloroplast (ctpHRPC; Figure 1C); and (4) pTRAkc-ERH-HRPCΔNC encoding HRP C lacking its native N-terminal ER-targeting signal peptide and ctVSS but having ER targeting and retention signals fused to its 5′ and 3′ termini, respectively (erhHRPC; Figure 1D), for targeting of HRP C to the ER.

### 2.2. Screening of Crude Extracts for Recombinant HRP C Expression

Successful transient expression of HRP C in *N. benthamiana* leaves infiltrated individually with these 4 different constructs was assessed by observing the presence or absence of HRP C protein resolved by SDS-PAGE from crude leaf extracts and detected by western blotting using a polyclonal anti-HRP C antibody. A range of protein bands (35–46 kDa) were visualized in samples from leaves infiltrated with pTRAc-HRPC, pTRAc-HRPCΔC, and pTRAkc-ERH-HRPCΔNC at 3, 5, and 7 days post-infiltration (dpi). Figure 2 shows the multiple bands detected by western blot in leaf extracts infiltrated with these constructs and sampled at 5 dpi (lanes 1 to 3). The range of bands was comparable with those of a commercially available HRP C resolved similarly (Figure 2; lane +); recombinant HRP C protein expressed in BY2 cells has also been shown to be heterogeneous in size [10].

The sizes of the HRP C bands detected on western blots varied depending on the construct tested. When pTRAc-HRPC was used for expression, the majority of vHRPC comprised of a 42 kDa-sized protein with several smaller bands ranging from 34 kDa, as well as a higher 44 kDa band (Figure 2, lane 1). When HRP C was targeted to the apoplast using pTRAc-HRPCΔC, similar bands, as well as an additional larger band, were seen. The appearance of this larger band appears distorted by the presence of another protein (Figure 2, lane 2), but is approximately 46 kDa in size. HRP C targeted to the ER showed a similar 46 kDa-sized protein in addition to the others, also distorted by another protein (Figure 2, lane 3).

Samples from leaves infiltrated with pTRAc-CTP-HRPΔNC showed that very little HRP C protein accumulated at 1, 3, 5, and 7 dpi (Figure S1) when targeted to the chloroplast, and it was predominantly of one molecular weight (Figure 2; lane 4).

Samples from leaves infiltrated with the *Agrobacterium* strain LBANSs negative control showed no reaction on the western blots at any dpi (Figure 2; lane −), indicating that the anti-HRP C antibody reacted specifically with recombinantly expressed HRP C and not with any native *N. benthamiana* proteins.

Similar results to those observed above were seen with samples that were co-infiltrated with the silencing suppressor (pBIN-NSs) from the tomato spotted wilt virus (TSWV). Although this construct has the potential to enhance transient protein expression by suppressing host post-transcriptional silencing [24], significant qualitative increases in HRP C expression levels were not observed in leaf extracts sampled within the first 7 dpi (Figure S1).

### 2.3. Peroxidase Assays on Crude Extracts

Preliminary quantitative peroxidase enzyme assays of all extracts sampled from 3 to 7 dpi confirmed the presence of active HRP C for vHRPC and aHRPC, which ranged from 50 to 80 U/g fresh weight plant material. Interestingly, cptHRPC did not show any activity, which is not surprising since very little cptHRPC was expressed (Figure S1). These preliminary tests indicated that pTRAc-HRPC and pTRAc-HRPCΔC constructs generated the highest levels of HRP C. Further peroxidase assays on crude extracts harvested from two whole plants infiltrated with these constructs at 3, 5, and 7 dpi were repeated four times, and results are shown in Figure 3. HRP C activity ranged from approximately 30–50 U/g fresh weight plant material, depending on the sampling day and which construct was tested. vHRPC showed consistently higher activity per gram of tissue when compared to levels of aHRPC, and both samples showed highest activity at 5 dpi. vHRPC had an activity of 50.6 ± 4.0 U/g at 5 dpi, which decreased slightly to 41.5 ± 4.0 U/g at 7 dpi. aHRPC had an activity of 39.6 ± 3.3 U/g at 5 dpi, which decreased to 33.8 ± 4.6 U/g at 7 dpi. Control plants infiltrated with the silencing suppressor construct showed only negligible peroxidase activity at the dilution used. The construct expressing vHRPC was thus selected for subsequent use in generating larger amounts of HRP C in order to optimize a purification protocol for the protein.

### 2.4. Purification of HRP C

**Figure 4:**
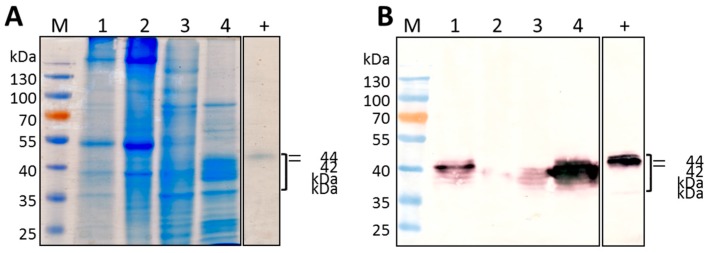
SDS-PAGE and Western blot analysis of total soluble protein fractions of HRP C. (**A**) Coomassie-stained SDS-polyacrylamide gel and (**B**) the corresponding western blot using anti-HRP antibody of ammonium sulphate fractions of total soluble protein extracted from vHRPC-expressing *N. benthamiana*. Lanes: Prestained protein ladder (M); total soluble protein extract from leaf tissue (1); 0–40% fraction (2); 40–60% fraction (3); 60–80% fraction (4); commercial Type I HRP C (400 mU) (+).Equal amounts of total soluble protein (8.8 µg) were loaded in each of the lanes (2–4). The bracket indicates the area in which HRP C specific bands can be seen (35 to 44 kDa).

Ammonium sulphate fractionation was carried out as an initial step towards purifying vHRPC from 12.5 g of leaves infiltrated with pTRAc-HRPC. Coomassie blue staining of the 4 fractions separated on a SDS-PAGE gel (Figure 4A) and subsequent western blot analysis (Figure 4B) showed the majority of vHRPC (70%) was present in the 60–80% fraction (lane 4), while the remaining 30% of vHRPC was present in the 40–60% fraction (lane 3). Peroxidase activity assays of each fraction substantiated these results, with the highest levels measured in the 60–80% fraction, lower levels in the 40–60% fraction, and negligible peroxidase activity associated with the 0–40% fraction. Consequently, HRPC collected in the 40–80% ammonium sulphate fractionation step was included in the protocol for further downstream purification.

**Figure 5:**
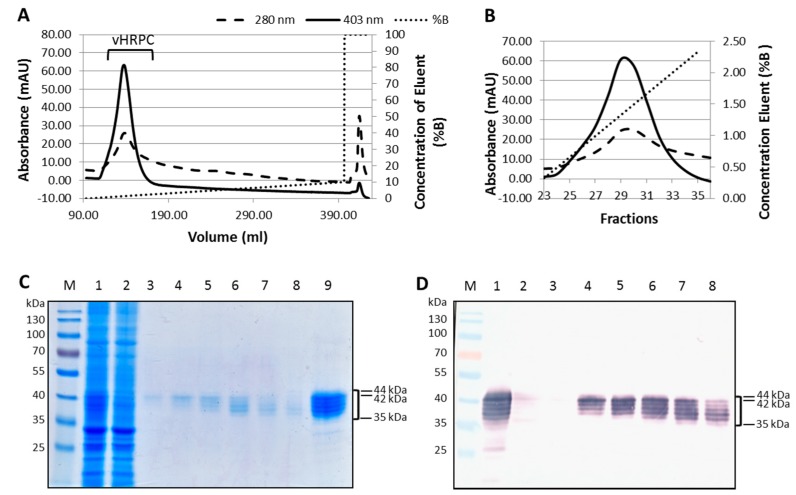
Purification of vHRPC using ion exchange chromatography (IEC). Chromatographic trace showing vHRPC elution (**A**) and fractions (**B**) from the IEC column with increasing salt concentration (% B). Coomassie blue staining (**C**) and western blot analysis (**D**) of IEC fractions and concentrated vHRPC purified by IEC. Lanes: Prestained protein ladder (M); fraction loaded onto column (1); unbound fraction (2); eluted fractions 26–31 (3–8, respectively); concentrated vHRPC (9). Bracket indicates HRP C.

vHRPC was easily traced during the chromatographic step by monitoring the 403 nm absorbance reading indicative of haem content (Figure 5A). Under optimized conditions (5 mM sodium acetate buffer, pH 5.6), vHRPC adsorbed to the cation exchange column, and negligible levels of vHRPC were found in the unbound fraction (Figure 5B,C, lane 2) compared to the total amount loaded onto the column (Figure 5B,C, lane 1). Figure 5A shows that vHRPC was successfully eluted by employing an increasing salt concentration gradient (% B, dotted line), as is visible by the 403 nm peak corresponding with a 280 nm peak (indicated by the bracket). All of the vHRPC was eluted when the salt concentration reached 20 mM NaCl (2% B, Figure 5A). Fractions from this peak separated by SDS-PAGE and analysed by western blot clearly show the presence of vHRPC (Figure 5C,D, lanes 4–8). The fractions from this peak were pooled and concentrated, and the concentrated vHRPC subsequently analysed by SDS-PAGE Coomassie staining and western blot (Figure 5C,D lane 9). Purified recombinant HRP C was heterogeneous, with the majority of vHRPC having a Mr range of approximately 35–44 kDa, which is consistent with the nature of the recombinant vHRPC in the crude extract. The specific activity of the concentrated vHRPc was measured by peroxidase assay to be 458 U/mg protein.

The extent of purification achieved using a combination of ammonium sulphate precipitation, IEC, and concentration is summarised in the purification table (Table 2): large scale batch ammonium sulphate precipitation of vHRPC yielded approximately 88% of the vHRPC present in the crude extract, resulting in a 4.4 fold increase in purity. Our calculations show that the majority of contaminating proteins were removed at the IEC step. The process of ammonium sulphate fractionation of the crude plant extract followed by IEC yielded a final preparation with an overall yield of 54%, 67.6-fold purification, and a high specific activity of 458 U/mg of protein (Table 2).

## 3. Discussion

Cloning of HRP C-expressing constructs into 4 different plant expression vectors designed to target recombinant protein to the ER, vacuole, apoplast, or chloroplast, and testing for transient expression of HRP C by *Agrobacterium*-mediated infiltration of the constructs into *N. benthamiana*, resulted in HRP C being successfully produced using all except the chloroplast-targeting construct, which resulted in negligible expression. The multiple HRP C-specific protein bands seen after electrophoretic fractionation of the various constructs is most likely related to the level and type of glycosylation of the recombinant protein occurring at a particular sampling time point. Mature HRP C has 8 occupied N-glycosylation sites [4], but it is possible that the differently sized bands represent incomplete stages of glycosylation of the protein in this case. In addition, proteins targeted to different cell compartments such as the ER and vacuole are often glycosylated differently [25], which may also explain the apparently heterogeneous nature of HRP C.

The construct pTRAc-HRPC should facilitate initial trafficking of HRP C into the ER, and from there into the vacuole under the influence of the native HRP C signal peptides [10]. Native HRP C possesses a paucimannosidic-type N-glycan structure [26], characteristic of proteins found in the vacuole [25]. These types of glycans are smaller than the complex-type N-glycans on proteins which are secreted to the apoplast [27]. Consequently, this could explain the larger 46 kDa-sized protein seen when HRP C was targeted to the apoplast using pTRAc-HRPCΔC, or localised in the ER after expression by pTRAkc-ERH-HRPCΔNC kDa, compared to the 44 kDa vHRPC, which is transported to the vacuole (Figure 2, lane 3). ER retention also appeared to have no significant increase or decrease on erhHRPC accumulation.

In the case of expression using the chloroplast-targeting construct, it is possible that much of the HRP C was degraded prior to being transported into the chloroplast, whereas the other recombinant proteins are targeted co-translationally directly into the ER, in which they were presumably protected from degradation. The size of the chloroplast-targeted protein is approximately 35 kDa, which corresponds to the size of unmodified HRP C [9]. This was not unexpected, as HRP C expression requires ER-targeting to initiate correct protein folding and processing, including glycosylation, haemin addition, disulphide bond formation, and Ca^2+^ incorporation [10,11]. This would have been facilitated by the native N-terminal HRP C signal peptide in both pTRAc-HRPC and pTRAc-HRPCΔC constructs, and the signal-peptide sequence from the murine *mAb24* heavy-chain gene in pTRAkc-ERH-HRPCΔNC. However, ER-directed protein processing is not facilitated by pTRAkc-rbcs1-cTP, which instead putatively targets the protein to the chloroplast due to the presence of the chloroplast-transit peptide from the potato *rbcS1* gene (pTRAc-CTP-HRPCΔNC construct).

Leaves infiltrated with the vacuole-targeting construct vHRPC showed consistently higher activity per gram of tissue when compared to levels expressed from the other constructs, and this was consequently selected for further experimentation. Ammonium sulphate precipitation of crude *N. benthamiana* leaf extracts indicated that the majority of the vHRPC was in the 60 to 80% fraction; subsequent cation exchange chromatography and concentration of HRP C-containing fractions resulted in a preparation having a specific activity of 458 U/mg of protein. A higher specific activity of approximately 1000 U/mg of protein has previously been obtained for recombinant HRP C expressed in *P. pastoris* [28], although direct comparison of the specific activity obtained in this study is not strictly correct since activity units have been differently defined. In addition, the HRP C produced in *P. pastoris* is hyperglycosylated, having a molecular mass of 65 kDa. The altered biochemical properties make it less amenable to purification by a similar strategy to that used for native HRP C [14]. Three successive chromatographic steps were carried out in the *P. pastoris* system [28] in order to obtain a product with such a high specific activity, thereby significantly increasing the downstream cost of production. However, in our system, only one chromatographic step was required to achieve a preparation with a high specific activity. Thus, production of HRP C in *N. benthamiana* has a significant advantage when compared to production in the *Pichia*-based system. Moreover, the specific activity of commercially available HRP C ranges from 50 to 350 U/mg, which is lower than the plant-produced HRP C in this study, making ours more commercially appealing [23].

The approximate HRP C content in crude leaf tissue was calculated to be 133 mg/kg. These levels produced are similar to the highest levels produced in insect larvae [17], and are higher than those attained from transgenic BY2 cells [12]. Measurements of the absorbance of purified vHRPC at 403 and 275 nm resulted in the calculation of an RZ value of >3. This indicates a high level of purity after just three purification steps. Furthermore, the use of transient expression technology rather than stable expression technology in this case also allows for the rapid production of raw material containing high levels of HRP C in a matter of just a few days, should it be required.

Although final yields recovered from IEC (54%) were low compared to those obtained using the yeast expression system, further optimization of the process would probably result in higher yields of pure HRP C. As it is, yields of recombinant HRP C achieved in this study are the highest levels reported in plant systems to date, and are comparable with the highest levels obtained in insect larvae.

A techno-economic analysis on the production of HRP C using transient expression system in *N. benthamiana* has been published based on the work described in this manuscript [23]. The analysis showed that HRP C production would be competitive at scales of more than 5 kg/year. However, increasing yields could reduce the cost and make it more competitive at scales of less than 5 kg HRP C/year. Such cost savings could be introduced by improving greenhouse productivity or making improvements to enzyme efficacy or stability by optimisation of constructs to allow additional increases in HRP C levels in crude leaf extracts, which could result in higher overall yields.

One of the most attractive advantages of using plants for recombinant protein production is that lower raw material costs translate to a lower cost of production [23]. Indeed, Nandi et al. [29] have developed a model to evaluate the transient expression of recombinant proteins in *N. benthamiana*. Techno-economic analysis of the production of monoclonal antibodies using this model shows that there is a significant reduction in capital investment and cost of goods compared with those of a mammalian cell production platform on a similar scale. Transient expression of HRP C in *N. benthamiana* may therefore prove to be a more cost effective method of production when compared to using insect cell production systems, even when similar yields (g/kg) are obtained. In addition, the continual availability of *N. benthamiana* all year round for HRP C production is preferable and more commercially viable than having to rely on the seasonal availability of *A. rusticana* crops.

## 4. Materials and Methods

### 4.1. Gene Synthesis

A synthetic *Nicotiana benthamiana* codon-adapted *HRP C* gene (353 aa), based on the amino acid sequence published by Welinder [9] (UniProt: P00433 (PER1A_ARMRU)), was synthesized (GENEART, Regensburg, Germany). In addition, a C-terminally truncated gene lacking the C-terminal-dependent vacuolar sorting signal (ctVSS, 339–353 aa) and N- and C-terminally truncated variant versions of the *HRP C* gene lacking both the ctVSS (339–353 aa) and the N-terminal signal sequence (1–30 aa), referred to as *HRPCΔC* and *HRPCΔNC*, respectively, were also synthesized (Table 1, Figure 1). Specific restriction enzyme sites were added during gene synthesis to facilitate cloning where possible.

### 4.2. Construction of Plant Expression Vectors

*HRP C* genes were cloned into the pTRA binary plant expression vectors provided by Dr Rainer Fischer (Fraunhofer Institute, Aachen, Germany). Details of these vectors have been previously described by Maclean et al. [30] and Meyers et al. [20].

The full length *HRP C* and the C-terminally truncated *HRPCΔC* genes were cloned into the destination vector pTRAc, resulting in the recombinant constructs pTRAc-HRPC and pTRAc-HRPCΔC, respectively (Table 1). The N- and C-terminally truncated *HRPCΔNC* was cloned into the destination vector pTRAkc-rbcs1-cTP, resulting in pTRAc-CTP-HRPCΔNC, which targets the product to the chloroplast (Table 1). *HRPCΔNC* was PCR amplified using primers hrpERH-F 5′, CTGTCATGAACCTTACTCCTACCTTCTACG 3′, and hrpERH-R 5′ ATAGCGGCCGCAGAGTTGCTGTTCACC 3′ in order to add *Bsp*HI and *Not*I sites to the respective termini of the gene. The amplified product was restricted with *Bsp*HI/*Not*I and cloned into the *Nco*I and *Not*I sites of pTRAkc-ERH, resulting in pTRAkc-ERH-HRPCΔNC (Table 1). This vector has the plant codon-optimized signal-peptide sequence from the murine *mAb24* heavy-chain gene, and a SEKDEL ER-retention signal, and thus should direct recombinant protein secretion to the ER where it should be retained. All recombinant vector construction was confirmed by restriction digests, PCR, and sequence analysis where relevant.

### 4.3. Agrobacterium tumefaciens-Mediated Transient Expression

Recombinant HRP C constructs (Table 1) were electroporated into electrocompetent *A. tumefaciens* GV3101::pMP90RK as described by Maclean et al. [30]. Recombinant *A. tumefaciens* were selected on LB agar containing rifampicin (50 µg/mL), kanamycin (30 µg/mL), and carbenicillin (50 µg/mL).

Recombinant *Agrobacterium*-HRP C strains were grown overnight in induction medium (LB broth containing 10 mM MES pH 5.6 and 20 µM acetosyringone) supplemented with antibiotics, as described previously, at 27 °C with agitation. For infiltration, cells were collected by centrifugation (4000× *g*) and resuspended in infiltration medium (10 mM MES, 10 mM MgCl_2_, 200 µM acetosyringone, 2% sucrose, pH 5.6). *Agrobacterium*-HRP C strains were infiltrated either separately or in combination with *A. tumefaciens* LBA4404 containing a silencing suppressor (pBIN-NSs) (provided by Marcel Prins, Laboratory of Virology, Wageningen, The Netherlands) from the tomato spotted wilt virus (TSWV). *A. tumefaciens* LBA4404 (pBIN-NSs) was cultured overnight at 27 °C with agitation in induction medium supplemented with rifampicin (50 µg/mL), kanamycin (30 µg/mL), and 2 mM MgSO_4_, and collected as described previously. *Agrobacterium* strains were diluted to an OD_600_ of 0.25 in infiltration medium. However, when *Agrobacterium*-HRP C strains were combined together with LBA4404 (pBIN-NSs), strains were diluted with infiltration medium to an OD_600_ of 0.5 and equal volumes added when combined (i.e., final OD_600_ of 0.25 for each strain). Diluted *Agrobacteria* suspensions were incubated at 22 °C for 2 h prior to infiltration directly into the abaxial air spaces of *N. benthamiana* leaves using a syringe. Plants infiltrated with LBA4404 (pBIN-NSs) served as a negative control in order to show that the anti-HRP C mouse polyclonal antibody reacted specifically with recombinantly expressed HRP C and not with other *N. benthamiana* proteins. Infiltrated plants were grown at 22 °C under a 16 h/8 h light/dark cycle at a light intensity of 60–80 µE/m^2^/s.

Large scale infiltrations were carried out by vacuum infiltration of whole *N. benthamiana* plants as described by Maclean et al. [30], and grown as previously described.

### 4.4. Time Trial Expression Studies

Expression of HRP C using the various constructs was confirmed and optimised by means of time trial expression studies. Three leaf discs cut using the lid of a 1.5 mL microfuge tube were harvested from agroinfiltrated leaves of each plant at 1, 3, 5, and 7 dpi. Leaf discs were ground in liquid nitrogen and soluble proteins extracted by the addition of 150 µL 1 × phosphate-buffered saline (PBS). The extract was centrifuged at 15,000× *g* for 5 min and the supernatant collected. HRP C presence was evaluated by means of western blotting.

### 4.5. Protein Analysis

Crude plant extract or protein samples were mixed with SDS sample application buffer [31], heated at 90 °C for 5 min, and separated on 10% SDS-PAGE gels. SDS-PAGE gels were either stained with Coomassie Blue G250 or transferred to nitrocellulose membrane by semi-dry electroblotting (Bio-Rad, Hercules, CA, USA). HRP C protein was detected with anti-HRP C mouse polyclonal antibody (1:5000; Abcam^®^, Cambridge, UK) in conjunction with goat anti-mouse-alkaline phosphate conjugate (1:7000; Sigma, St Louis, MO, USA). Detection was performed with NBT/BCIP (KPL, Washington, D.C., MD, USA). Type I HRP C (Sigma) served as the positive control.

Protein concentrations were determined using the Bio-Rad *DC* protein assay according to manufacturer’s instructions. Total protein concentrations in crude extracts and of purified HRP were obtained by using calibrated curves constructed using BSA and HRP (Sigma) as standards, respectively.

The Reinheitszahl (RZ) value is a measure of hemin content and is defined as the ratio of the absorbances at 403 and 275 nm (*A*_403_/*A*_275_) [6].

### 4.6. Peroxidase Assay

Enzyme activity was determined by assessing guaiacol oxidation in a reaction mixture containing 300 µM guaiacol and 130 μM hydrogen peroxide in 100 mM potassium phosphate buffer, pH 7.0. Activity levels were determined by using calibrated curves constructed using known amounts of commercially available HRP C (Sigma and Faizyme, Cape Town, South Africa). 1 Unit of HRP C is defined as the amount of enzyme required to catalyse the conversion of 1 micromole of hydrogen peroxide per minute at 25 °C.

In the case of crude extract testing, samples were diluted either 10 or 50 fold depending on the construct and time point sampled. Control plants infiltrated with the silencing suppressor construct only served as a negative control for crude extract testing. The native peroxidase level obtained from control plants at these dilutions was of a negligible amount.

### 4.7. Protein Extraction and Purification

All operations were carried out at 4 °C unless otherwise stated.

#### 4.7.1. Crude Sample Preparation

*N. benthamiana* plants expressing vHRPC were harvested either 5 or 7 dpi. Leaf tissue was homogenized directly after harvesting with ice cold 0.1 M potassium phosphate buffer (pH 7) at a ratio of 1:4 (mass:vol) for approximately 10 min. The crude extract was then incubated on ice for 20 min before being centrifuged at 13,000× *g* for 15 min at 4 °C. The supernatant was filtered through 3 layers of Miracloth™ and stored on ice. The remaining homogenized tissue was re-extracted with ice cold 0.1 M potassium phosphate buffer (pH 7) at a ratio of 1:1. The re-extracted sample was processed as previously described. Extracted samples were pooled and subjected to ammonium sulphate (NH_4_)_2_SO_4_ fractionation. A mass of 20 g leaf tissue resulted in approximately 100 mL of crude sample extract.

#### 4.7.2. Ammonium Sulphate Fractionation

The crude extract was brought to 40% (NH_2_)_2_SO_4_ saturation by the slow addition of powdered (NH_4_)_2_SO_4_ with stirring on ice. The solution was allowed to equilibrate for 1 h on ice before the precipitate was pelleted by centrifugation at 10,000× *g* for 30 min. The supernatant was collected and brought to 80% (NH_2_)_2_SO_4_ saturation as previously described. The precipitate was collected as before and dissolved in 5 mM potassium phosphate buffer, pH 7.0 before being dialysed against three changes of 5 mM sodium acetate buffer, pH 5.6 (buffer A).

#### 4.7.3. Cation Exchange Chromatography

An ÄKTA explorer (GE Healthcare Life Sciences, Little Chalfont, Buckinghamshire, UK) was used to perform ion exchange chromatography (IEC). The dialysed (NH_2_)_2_SO_4_ fraction was loaded onto a HiPrep SP XL column (GE Healthcare Life Sciences, 20 mL bed volume), pre-equilibrated with buffer A. The column was washed with 100 mL of buffer A at a flow rate of 5 mL/min. vHRPC was eluted using an increasing concentration (0–10%) of 1 M NaCl in 5 mM sodium acetate buffer, pH 5.6 (buffer B). Protein and haem content of the 5 mL fractions collected over this gradient elution were monitored by absorbance at 280 nm and 403 nm, respectively. Fractions were also tested for vHRPC content by peroxidase assay. Fractions showing significant HRP activity were pooled and concentrated using an Amicon^®^ Ultra centrifugal filter (Merck, Darmstadt, Germany).

## Figures and Tables

**Table 1:** Plant HRP C expression vectors constructed using synthetic *HRP C* genes.

Construct	Vector	Insert ^b^	Signal Peptide	Recombinant ^b^ Protein Name	Cellular Location ^a^
pTRAc-HRPC	pTRAc	*HRPC*	5′ HRP C native 3′ Vacuolar sorting signal	vHRPC	vacuole
pTRAc-HRPCΔC	pTRAc	*HRPCΔC*	5′ HRP C native No vacuolar sorting signal	aHRPC	apoplast
pTRAc-CTP-HRPCΔNC	pTRAkc-rbcs1-cTP	*HRPCΔNC*	5′ Potato *rbcS1*	ctpHRPC	chloroplast
pTRAkc-ERH-HRPCΔNC	pTRAkc-ERH	*HRPCΔNC*	5′ Murine mAb24 heavy-chain 3′ KDEL ER retention signal	erhHRPC	endoplasmic reticulum

^a^ Theoretical cellular location of HRP C based on the signal sequences in the constructs; ^b^ Schematic representation of the insert, signal peptides and protein identity are given in Figure 1.

**Table 2:** Purification of vHRPC from infiltrated *Nicotiana benthamiana* leaves.

Purification Step	Specific Activity ^a^ (U·mg^-1^ Protein)	Yield (%)	Purification Fold
Crude	6.78	100	1
40–80% Ammonium sulphate	29.82	87.8	4.4
Ion exchange chromatography	458.17	54	67.6

^a^ Unit of HRP C is defined as the amount of enzyme required to catalyse the conversion of 1 micromole of hydrogen peroxide per minute at 25 °C.

## Supplementary Materials

Figure S1 is available online at http://www.mdpi.com/1422-0067/19/1/115/s1.
