# Supplementary material for: Transient Expression and Purification of Horseradish Peroxidase C in Nicotiana benthamiana
Source: Int J Mol Sci. 2018 Jan 1;19(1):115. doi: 10.3390/ijms19010115 (PMC5796064; doi:10.3390/ijms19010115)
Supplement: Supplementary file 1 [file ijms-19-00115-s001.pdf]

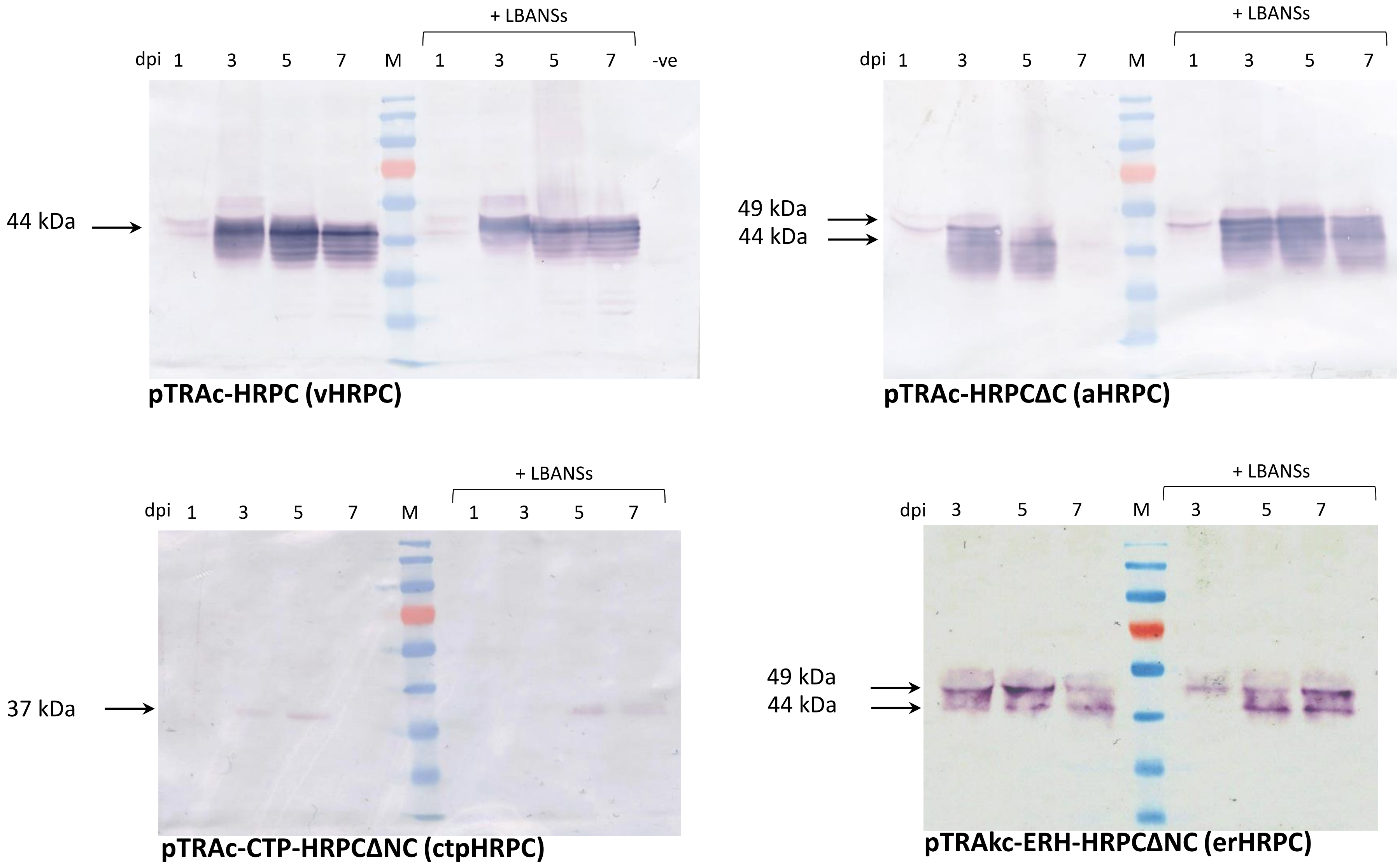

Figure S1. Western blots of HRP C in crude extracts from leaves infiltrated with engineered constructs. HRP C was detected using mouse polyclonal anti-HRP C. Crude leaf extracts were analysed from plant tissue infiltrated with recombinant Agrobacterium strains carrying the following expression vectors pTRAc-HRPC (vHRPC; pTRAc-HRPC Δ C (aHRPC); pTRAkc-ERH-HRPC Δ NC (erhHRPC) and pTRAc-CTP-HRPC Δ NC (ctpHRPC) and harvested on 1, 3, 5 and 7 days post infiltration (dpi). +LBANSs: crude extracts from leaves co-infiltrated with LBANSs and harvested on the same dpi. Prestained protein ladder (M).
